# Non-Pathological Opacification of the Cavernous Sinus on Brain CT Angiography: Comparison with Flow-Related Signal Intensity on Time-of-Flight MR Angiography

**DOI:** 10.3390/healthcare9010094

**Published:** 2021-01-18

**Authors:** Sun Ah Heo, Eun Soo Kim, Yul Lee, Sang Min Lee, Kwanseop Lee, Dae Young Yoon, Young-Su Ju, Mi Jung Kwon

**Affiliations:** 1 Department of Radiology, Hallym University Sacred Heart Hospital, College of Medicine, Hallym University, Seoul 14068, Korea; hsa0528@hallym.or.kr (S.A.H.); leeyul210@naver.com (Y.L.); twin393@hanmail.net (S.M.L.); kwanseop@hallym.or.kr (K.L.); 2 Department of Radiology, Kangdong Sacred Heart Hospital, College of Medicine, Hallym University, Seoul 14068, Korea; evee0914@chollian.net; 3 National Medical Center, Seoul 04564, Korea; juyoungsu.zorro@gmail.com; 4 Department of Pathology, Hallym University Sacred Heart Hospital, College of Medicine, Hallym University, Seoul 14068, Korea; mulank99@hallym.or.kr

**Keywords:** cavernous sinus, magnetic resonance angiography, computed tomography angiography, brain, consensus

## Abstract

*Purpose*: To investigate the non-pathological opacification of the cavernous sinus (CS) on brain computed tomography angiography (CTA) and compare it with flow-related signal intensity (FRSI) on time-of-flight magnetic resonance angiography (TOF-MRA). *Methods*: Opacification of the CS was observed in 355 participants who underwent CTA and an additional 77 participants who underwent examination with three diagnostic modalities: CTA, TOF-MRA, and digital subtraction angiography (DSA). Opacification of the CS, superior petrosal sinus (SPS), inferior petrosal sinus (IPS), and pterygoid plexus (PP) were also analyzed using a five-point scale. The Wilcoxon test was used to determine the frequencies of the findings on each side. Additionally, the findings on CTA images were compared with those on TOF-MRA images in an additional 77 participants without dural arteriovenous fistula (DAVF) using weighted kappa (κ) statistics. *Results*: Neuroradiologists identified non-pathological opacification of the CS (*n* = 100, 28.2%) on brain CTA in 355 participants. Asymmetry of opacification in the CS was significantly correlated with the grade difference between the right and left CS, SPS, IPS, and PP (*p* < 0.0001 for CS, *p* < 0.0001 for SPS, *p* < 0.0001 for IPS, and *p* < 0.05 for PP). Asymmetry of the opacification and FRSI in the CS was observed in 77 participants (CTA: *n* = 21, 27.3%; TOF-MRA: *n* = 22, 28.6%). However, there was almost no agreement between CTA and TOF-MRA (*κ* = 0.10, 95% confidence interval: −0.12–0.32). *Conclusion*: Asymmetry of non-pathological opacification and FRSI in the CS may be seen to some extent on CTA and TOF-MRA due to anatomical variance. However, it shows minimal reliable association with the FRSI on TOF-MRA.

## 1. Introduction

The cavernous sinus (CS) is one of the dural venous sinuses within the human cranium [1]. The CS receives blood from the superior and inferior ophthalmic veins and the superficial cortical veins and is continued to the basilar plexus of veins posteriorly [2,3]. The CS drains by two larger channels, the superior petrosal sinus (SPS) and inferior petrosal sinus (IPS), and finally into the internal jugular vein via the sigmoid sinus, anastomosing with the PP by way of the sphenoid emissary veins [2,3]. However, it is rarely seen on magnetic resonance venography in healthy subjects, except in the presence of diseases involving the CS. Computed tomography angiography (CTA) is commonly regarded as an excellent method for evaluating intracranial arterial abnormalities, with the advantages of being a minimally invasive procedure and providing good resolution. Recently, multidetector CTA has successfully replaced diagnostic digital subtraction angiography (DSA) in the evaluation of various vascular lesions [4,5,6]. CTA is better than time-of-flight magnetic resonance angiography (TOF-MRA) in evaluating the size and location of the carotid cavernous fistula (CCF) [7].

When reading CTA, we often observe one CS with asymmetric bulging contour or higher opacification than on the other side of CT source data. In many cases, the presence of a lesion is often determined using only a single CTA. This finding may be a false positive lesion mimicking dural arteriovenous fistula (DAVF) or CCF. The flow-related signal intensity (FRSI) due to venous regurgitation in the cavernous sinus is often seen on TOF-MRA [3,8]. We would like to investigate the relationship between the non-pathologic opacification of the cavernous sinus in brain CTA and whether FRSI on TOF-MRA can appear the same in the same place. The purpose of our study was to investigate the asymmetry of opacification in the CS and the relatively higher opacification of one side of the patients’ brain CTA and to compare it with FRSI on TOF-MRA in patients without DAVF in the CS.

## 2. Methods

### 2.1. Patient Selection

This study conformed to the principles of the Helsinki Declaration of 1975 (revised version 2013). The study protocol was approved by the Institutional Review Board of Hallym University Sacred Heart hospital (No. 2019-09-027-002), which waived the need for written informed consent from the subjects due to the retrospective nature of the study.

A computerized search of the radiology database at this hospital for a 6-month period from May 2017 to October 2017 revealed data for 690 patients who underwent brain CTA. A total of 335 patients were excluded on the basis of the following criteria: aneurysm (*n* = 223), craniotomy (*n* = 67), trauma (*n* = 16), large territory infarction (*n* = 4), arteriovenous malformation (*n* = 4), tumor (*n* = 3), DAVF (*n =* 2), and poor image quality (*n* = 16). Of the patients with DAVF, the fistulas were located in the transverse–sigmoid sinus in two patients. Patients with chronic small infarctions (small cerebellar infarctions or lacunar infarcts) and small punctate hemorrhages (microbleeds) were included. A total of 355 patients (173 men and 182 women) were evaluated. They had no clinical symptoms, signs, and CTA findings suggestive of DAVF, such as superior ophthalmic vein and/or orbital or periorbital soft tissue swelling. The mean age of the study group was 56 years (standard deviation: 16 years).

Next, we performed to identify patients who underwent CTA, TOF-MRA, DSA, and 4-vessel cerebral angiography from September 2010 to September 2017. This cohort of 144 patients was used to further filter patients who (1) had no lesions with arteriovenous fistula and (2) who underwent brain CTA and time-of-flight magnetic resonance angiography (TOF-MRA) with 3-tesla magnetic resonance imaging (3T MRI) within 3 months before or after DSA. A total of 77 participants (35 men and 42 women) met these criteria. The specific diagnoses made according to the DSA images were as follows: aneurysm in 43 patients, cerebral artery or carotid artery stenosis in 20 patients, intra-axial tumor in three patients, extra-axial tumor in three patients, vertebral artery dissection in three patients, and normal status in five patients. All included cases had undergone pretreatment CTA and TOF-MRA. The mean age of the study group was 62 years (standard deviation: 13 years).

### 2.2. Image Acquisition

CTA was performed with a 128-section CT system Somatom Definition Flash 256, EDGE 128, Siemens Healthineers (Erlangen, Germany). A summary of the CTA imaging parameters is shown in Table 1. 

Contrast medium with an iodine concentration of 350 or 370 mg/mL was injected into the antecubital vein through an 18- or 20-gauge intravenous cannula followed by a saline chaser bolus. MRI was performed using a 3T MRI system (Achieva; Philips Healthcare, Best, the Netherlands) with a 32-channel head coil. We performed the intracranial 3D TOF-MRA protocol in all participants using the following parameters: repetition time, 23 ms; echo time, 3.5 ms; and a tilted optimized non-saturating excitation pulse with a central flip angle of 20°. The section thickness was 1.2 mm. A field of view of 200 × 200 mm^2^ was used with a matrix of 500 × 307 and a single excitation. The acquisition time was 4 min 12 s. The angiography procedure was standardized by typically placing 5F or 6F catheters into the internal carotid arteries or vertebral arteries through an artery in the groin; these were subsequently navigated to the neck vessels under fluoroscopy. Biplane DSA images of the entire circulation were obtained, and conventional 2D DSA was performed with an angiography system (Allura Xper FD20/20, biplane; Philips Healthcare, Best, the Netherlands). Arterial-to-venous-phase DSA images were obtained for all patients.

### 2.3. Image Interpretations

All 432 CTA (cohort 1 and 2) and 77 TOF-MRA (cohort 2) examinations were independently evaluated by two neuroradiologists (with 3 and 10 years of experience in neuroradiology), and the final judgments were obtained by consensus. The observers described the CT appearance of the CS according to the asymmetry of the CS and the opacification of the CS, SPS, IPS, and pterygoid plexus (PP). Optimal visualization of the CS, SPS, IPS, and PP was obtained with continuous opacification of both axial CTA source images. Hounsfield measurements were made for participants with a region of interest of 4–7 mm^2^ centered within the CS, SPS, IPS, PP, and transverse sinus. Three measurements of the mean attenuation were made in each sinus, and the average value was used for analysis.

**Figure 1:**
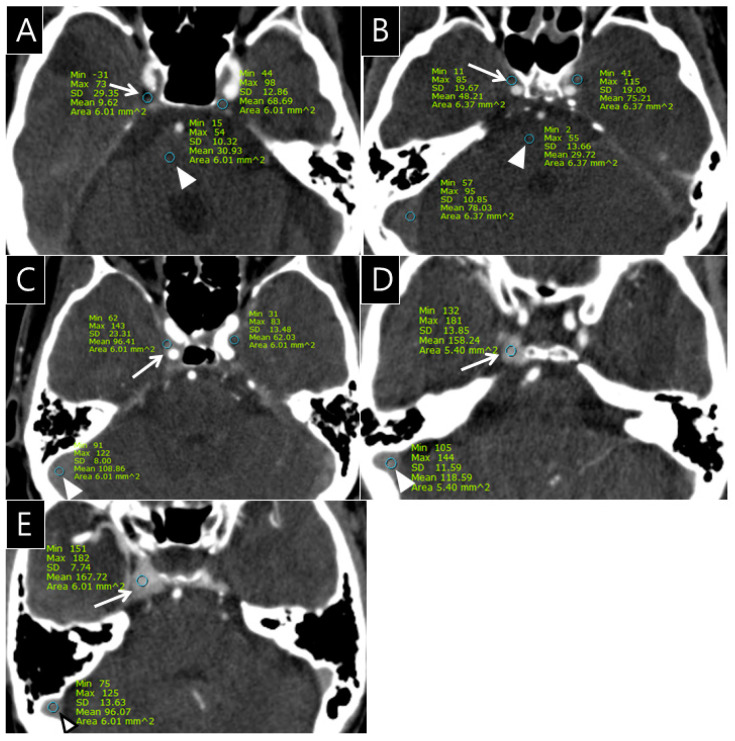
Examples of the 5-point grading scale for the cavernous sinus (CS) on computed tomography angiography (CTA); (**A**), Grade 0: Opacification of the right CS (arrow, CT number: 9.6 HU) is less than or equal to the density of the pons (arrowhead, CT number: 30.9 HU) on the CTA source image; (**B**), Grade I: Opacification of the right CS (arrow, CT number: 48.2 HU) is greater than the density of the pons (arrowhead, CT number: 29.7 HU) on the CTA source image; (**C**), Grade II: Opacification of the right CS (arrow, CT number: 96.4 HU) is less than or equal to that of transverse sinus (arrowhead, CT number: 108.8 HU) on the CTA source image; (**D**), Grade III: Opacification of the right CS (arrow, CT number: 158.2 HU) is greater than that of the transverse sinus (arrowhead, CT number: 118.6 HU) in less than or equal to one-third of the CS; (**E**), Grade IV: Opacification of the right CS (arrow, CT number: 167.7 HU) is seen in more than one-third of the CS (arrowhead, CT number of transverse sinus: 96.1 HU).

The radiologists quantitatively rated the opacification observed in the CS, SPS, IPS, and PP on the 432 CTA images (cohorts 1 and 2) using a 5-point grading scale as follows: grade 0 = the opacification of the sinus is less than or equal to the density of pons; grade I = the opacification of the sinus is above the density of pons; grade II = the opacification of the sinus is less than or equal to that of the transverse sinus; grade III = the opacification of the sinus is more than that of the transverse sinus in less than or equal to one-third of the CS; and grade IV = the opacification of the CS is seen in more than one-third of the CS (Figure 1).

**Figure 2:**
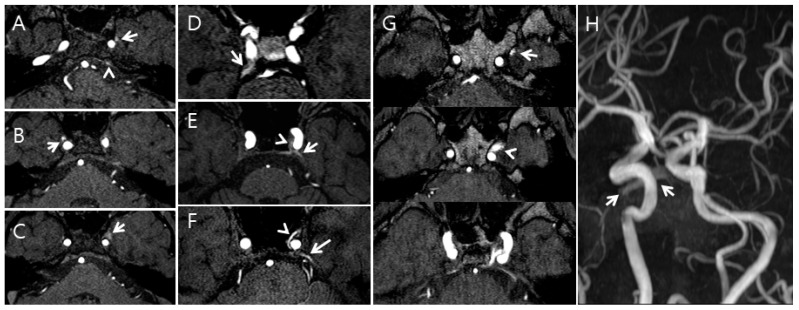
Examples of the five-point grading scale for the cavernous sinus (CS) on time-of-flight magnetic resonance angiography (TOF-MRA); (**A**), Grade I: The left pterygoid sinus flow-related signal intensity (FRSI) (arrow) and left inferior petrosal sinus FRSI (arrowhead) were greater than the signal intensity of the pons on the TOF-MRA source image; (**B**), Grade II: The right pterygoid sinus FRSI (arrow) is equal to that originating from arterial structures of the same size on the TOF-MRA source image; (**C**), Grade III: High signal intensity (arrow) is observed in less than or equal to one-third of the CS and pterygoid plexus on the TOF-MRA source image; (**D**), Grade II: The right superior petrosal sinus FRSI (arrow) is equal to that originating from arterial structures of the same size on the TOF-MRA source image; (**E**), Grade III: A high signal intensity (arrowhead) is seen in less than or equal to one-third of the CS, and the left superior petrosal plexus (arrow) is seen on the TOF-MRA source image; (**F**), Grade IV: A high signal intensity (arrowhead) is seen in more than one-third of the CS, and the superior petrosal plexus (arrow) is seen on the maximum intensity projection source image; (**G**,**H**), Grade IV: A high signal intensity (arrowhead) in more than one-third of the CS and pterygoid plexus (arrow) is seen on the maximum intensity projection source image (**G**). A high signal intensity (arrows) was seen in the pterygoid plexus on the TOF-MRA maximum intensity projection image (**H**).

Additionally, the radiologists qualitatively assessed the FRSIs observed in the CS, SPS, IPS, and PP on the TOF-MRA images (cohort 2) using a 5-point grading scale as follows: grade 0 = the FRSI of the CS, SPS, IPS, and PP is less than or equal to the signal intensity of pons; grade I = the FRSI of the SPS, IPS, and PP is above the signal intensity of pons; grade II = the FRSI of the CS, SPS, IPS, and PP is equal to that originating from arterial structures of the same size; grade III = a high signal intensity is seen in less than or equal to one-third of the CS, SPS, IPS, and PP; and grade IV = a high signal intensity is seen in more than one-third of the CS, SPS, IPS, and PP (Figure 2).

Of the TOF-MRA images, 77 obtained before or after DSA were independently evaluated by two neuroradiologists, and the final judgments were obtained by consensus. A total of 77 DSA images were simultaneously reviewed by radiologists to determine whether DAVFs were present at the CS.

### 2.4. Statistical Analysis

In 355 participants from cohort 1 and 77 participants from cohort 2, opacification of the CS, SPS, IPS, and PP on CTA and the FRSI were classified according to a 5-point scale, and their frequency was evaluated. The Wilcoxon test was used to determine whether the opacifications were more frequent on the left side or the right side in cohort 1. The degree of agreement between opacification of the CS, SPS, IPS, and PP on CTA and the FRSI on TOF-MRA was calculated using square-weighted κ values. We evaluated interobserver agreement between the two observers using κ statistics. All statistical analyses were performed using the statistical software package SPSS version 24.0. (SPSS Inc., IBM, Chicago, IL, USA). A *p* value of < 0.05 was considered statistically significant.

## 3. Results

**Figure 3:**
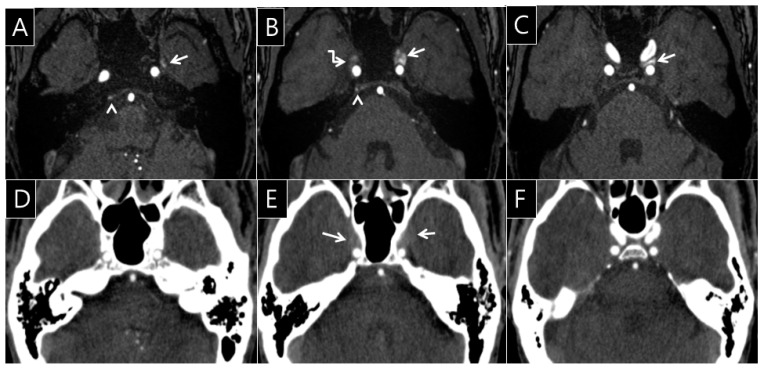
A 56-year-old man’s brain computed tomography angiography (CTA) and time of flight magnetic resonance angiography (TOF-MRA) images. On the TOF-MRA source images (**A**–**C**), the left pterygoid plexus (arrow, **A**) shows FRSI on TOF-MRA (**A**). The FRSI is contiguous into the left CS, occupying more than one-third of the CS on TOF-MRA (arrows, **B**, **C**, grade IV). Mild FRSI was observed in the right inferior petrosal sinus (arrowhead, **A**, **B**, grade I) and right CS (broken arrow, **B**, grade III). However, the opacification of the venous sinus is less than or equal to the density of pons (**D**, **F**, grade 0). The degree of opacification in the both CS is greater than the density of the pons in the same regions on brain CTA (arrows, **E**, grade I). Minimal agreement with the consensus score (*κ* = 0.30, 95% confidence interval: 0.10–0.51) is shown; Note: κ, degree of intra-observer agreement between CTA and TOF-MRA.

Of the 355 total CTA images from cohort 1, opacification of the right CS was seen in 182 patients (51.3%) and was rated as grade I in 77 patients (21.7%), grade II in 41 patients (11.6%), grade III in 37 patients (10.4%), and grade IV in 27 patients (7.6%). Opacification of the left CS was seen in 182 patients (51.3%) and was rated as grade I in 77 patients (21.7%), grade II in 36 patients (10.1%), grade III in 47 patients (13.2%), and grade IV in 22 patients (6.2%). The difference in the frequency of opacification between the right and left structures was not statistically significant (Table 2). The neuroradiologists identified asymmetry of the CS (*n* = 100, 28.2%) in 355 participants on brain CTA. Asymmetry of contrast opacification in the CS was statistically correlated with the grade difference between the right and left CS, SPS, IPS, and PP (*p* < 0.0001 for CS, *p* < 0.0001 for SPS, *p* < 0.0001 for IPS, and *p* < 0.05 for PP) (Table 3). Asymmetry of the CS was observed in 77 participants (CTA: *n* = 21, 27.3%; TOF-MRA: *n* = 22, 28.6%). However, there was almost no agreement between CTA and TOF-MRA (*κ* = 0.10, 95% confidence interval: -0.12~0.32). In the analysis of the 77 CTA and TOF-MRA images, opacifications of right SPS, right PP, and left PP on brain CTA and FRSI on TOF-MRA showed minimal agreement with the consensus score (*κ* = 0.23~0.30) (Table 4). The right SPS showed opacification (*n* = 39, 50.7%) and FRSI (*n* = 8, 10.4%). The opacification of the right SPS on brain CTA and FRSI on TOF-MRA showed minimal agreement with the consensus score (*κ* = 0.25, 95% confidence interval: 0.10~0.40). The right PP showed opacification (*n* = 18, 23.4%) and FRSI (*n* = 11, 11.7%), which showed minimal agreement with the consensus score (*κ* = 0.23, 95% confidence interval: ≈0.06–0.40). The left PP showed opacification (*n* = 21, 27.3%) and FRSI (*n* = 16, 20.8%). Opacifications of the right CS, right IPS, left CS, left SPS, and left IPS on the brain CTA and FRSI on TOF-MRA showed negligible agreement with the consensus score (κ = ≈0.06–0.15) (Table 4). Opacification of the left PP on brain CTA and FRSI on TOF-MRA showed minimal agreement with the consensus score (κ = 0.30, 95% confidence interval: ≈0.10–0.51) (Table 4, Figure 3). No DAVFs were seen in the CS on DSA images in any patients with FRSIs in the SPS, IPS, and PP. For rating the grade of the opacification and signal in the venous sinuses, the interobserver agreements between the two neuroradiologists were excellent with a consensus score of *κ* = 0.85 in cohort 1 and 0.84 in cohort 2, respectively. 

## 4. Discussion

The present study showed that the opacification and asymmetry of the CS can be observed on brain CTA without clinical symptoms or signs of DAVF. FRSI in the TOF-MRA source are often observed in the CS and the surrounding venous sinuses, which are associated with venous regurgitation and are less often associated with DAVF or CCF [3]. 

Watanabe et al. [3] reported that the FRSIs of the PP and CS were confirmed in 110 of 406 participants, of which 67 FRSIs were confirmed in PP only, and 43 FRSIs were confirmed in both PP and CS. The FRSI of PP appeared significantly higher on the left than on the right side, due to flow reversal in which the signal intensities of the left internal jugular vein and sigmoid sinus are higher than those of the right, suggesting that the pressure in the brachiocephalic vein may be the main reason [9]. The left brachiocephalic vein flowing into the superior vena cava passes through the anterior segment of the aortic arch and can be pushed between the sternum and the aortic arch, which explains the compression from an anatomical viewpoint [3,10]. In our study, the FRSIs in the right PP vs. left PP and right IPS vs. left IPS on TOF-MRA among 77 participants were 9 (11.69%) vs. 16 (20.78%) and 11 (14.29%) vs. 18 (23.38%), respectively. The FRSIs in the left PP and left IPS were higher than those in the right PP and IPS. However, these differences were not statistically significant in our study (Appendix A Table A1). 

We have speculated that asymmetric opacification of the CS may be associated with reversed venous flow, but the results showed that the FRSI on TOF-MRA was poorly correlated with asymmetric opacification of the CS. Asymmetric opacification of the CS only showed a distinct opacification between the artery and vein, and the morphology of the cavernous sinus; therefore, it can be inferred that it is difficult to show the correlation of the FRSI with TOF-MRA because of the poor temporal information in CTA. TOF-MRA and CTA techniques are inherently different. TOF-MRA, a completely different technique from CTA, is based on the principle of flow-related enhancement of stationary tissues in an imaged volume magnetically saturated by multiple repetitive radiofrequency (RF) pulses that drive down their steady-state magnetization levels. “Fresh” blood flowing into the imaged volume has not reached these pulses, and thus, it has high initial magnetization. Thus, the signal from inflowing blood appears paradoxically bright compared to that from the background tissue [11]. The use of a saturation method that suppresses craniocaudal flow is likely to be one of the potential reasons for the mismatch between CTA and TOF-MRA. The scan time of TOF-MRA is >4 min, even with the new version of the latest device that provides rich temporal information. Thus, it is difficult to obtain temporal information from CTA in a few seconds, and there is a limit to compare the flow on the same line as TOF-MRA. Therefore, venous flow reversal is unlikely to be reflected on CTA. 

Anatomic variations between the CS and surrounding venous structures can develop in the course of anastomosis development [12,13,14]. The superficial middle cerebral vein and sphenoparietal sinus are important venous structures serving as drainage routes from the cerebral hemisphere and meninges to the cervical venous system via the CS. In contrast to the superficial middle cerebral vein, the superior ophthalmic vein rarely forms an anatomic variation because it is a primitive vein that communicates with the prootic sinus, forming the CS [15]. We assume that the difference in the point of connection between the CS and the surrounding veins creates the asymmetry in CS structure. The CS is known to be anatomically bilaterally symmetric, but the transverse sinuses are commonly asymmetric, with the right transverse sinus being dominant in the majority of cases [16]. A unilateral atretic posteromedial segment of the left transverse sinus is also observed commonly [17]. This suggests that there may be a predominance of the CS showing normal variation with no functional problems and no asymmetry of the dural venous sinuses, such as the right dominant transverse sinus, sigmoid sinus, or internal jugular vein. Knowledge of the normal anatomical structure of the cerebral venous system and recognition of potential variations are essential to prevent the misinterpretation of CTA findings, not only for diagnosing diseases involving the CS but also for surgery and endovascular treatment of arteriovenous fistulas [18].

Our study had several limitations. First, the use of different CT systems in the case group may have caused heterogeneity in image quality and degree of opacification. Second, the brain CTA group in our study could not completely represent the normal population, even if they were selected as final participants, except those with major clinical illnesses. In 355 patients who underwent brain CTA, DAVF in the CS may not be completely ruled out based on clinical and CT findings alone. 

## 5. Conclusions

Asymmetric opacification of the CS shows a minimal reliable association with the FRSI on TOF-MRA in patients without DAVF in the CS. Asymmetry of the CS may be seen to some extent on CTA and TOF-MRA due to anatomical variance.

## Figures and Tables

**Table 1:** CTA imaging parameters.

CT Machines	Parameters
Somatom Definition Flash 256	FOV = 233 × 233 mm^2^; 128 × 0.6 collimation; 0.33 s rotation time; 2.57 s scan time; 0.7 pitch; 2 s delay; 120 kV tube voltage; 280 mA tube current; 0.6 mm section thickness; 0.4 reconstruction interval; caudocranial direction
Edge 128	FOV = 222 × 222 mm^2^; 128 × 0.6 collimation; 0.5 s rotation time; 2.67 s scan time; 1.2 pitch; 2 s delay; 120 kV tube voltage; 280 mA tube current; 0.6 mm section thickness; 0.4 reconstruction interval; caudocranial direction

CTA, computed tomography angiography; FOV, field of view.

**Table 2:** Frequency of opacification in the CS, SPS, IPS, and PP by grading in 355 participants (cohort 1).

Modality	Area	5-Point Scale					*p* Value *
		Grade 0 (%)	Grade I (%)	Grade II (%)	Grade III (%)	Grade IV (%)	
CT	R.CS	173 (48.7)	77 (21.7)	41 (11.6)	37 (10.4)	27 (7.6)	0.90
	L.CS	173 (48.7)	77 (21.7)	36 (10.1)	47 (13.2)	22 (6.2)	
	R.SPS	260 (73.2)	40 (10.3)	31 (8.7)	19 (5.4)	5 (1.4)	0.81
	L.SPS	264 (74.4)	31 (8.7)	40 (11.3)	16 (3.7)	4 (1.1)	
	R.IPS	310 (87.3)	30 (8.5)	10 (2.8)	3 (0.9)	2 (0.6)	0.42
	L.IPS	314 (88.5)	29 (8.2)	7 (2.0)	4 (1.1)	1 (0.3)	
	R.PP	337 (94.9)	7 (2.0)	5 (1.4)	3 (0.9)	3 (0.9)	0.45
	L.PP	335 (94.4)	9 (2.5)	4 (1.1)	4 (1.1)	3 (0.9)	

Notes: Data represent the number of cases. * Wilcoxon test was used to evaluate whether the finding was more frequent on the left or right side. However, the difference in frequency was not statistically significant. CT, computed tomography; CS, cavernous sinus; SPS, superior petrosal sinus; IPS, inferior petrosal sinus; PP, pterygoid plexus; R, right; L, left.

**Table 3:** Asymmetry of opacification in the cavernous sinus versus difference of CS, SPS, IPS, and PP by grading in 355 participants (cohort 1).

Asymmetry of CS	Estimate	Standard Error	*p* Value
vs. difference of CS	2.42	0.27	<0.0001
vs. difference of SPS	1.44	0.17	<0.0001
vs. difference of IPS	1.71	0.32	<0.0001
vs. difference of PP	0.67	0.26	0.01 (<0.05)

CS, cavernous sinus; SPS, superior petrosal sinus; IPS, inferior petrosal sinus; PP, pterygoid plexus.

**Table 4:** Frequency of opacification on brain CTA and flow-related signal intensity on TOF-MRA in the CS, SPS, IPS, and PP by grading in 77 participants (cohort 2).

Area	Modality	5-Point Scale					Degree of Intra-Observer Agreement Weighted Kappa Value (95% CI)
		Grade 0 (%)	Grade I (%)	Grade II (%)	Grade III (%)	Grade IV (%)	
R.CS	CT	25 (32.5)	14 (18.2)	12 (15.6)	18 (23.4)	8 (10.4)	0.06 (−0.05–0.17)
	MR	57 (74.0)	11 (14.3)	2 (2.6)	6 (7.8)	1 (1.3)	
R.SPS	CT	38 (49.4)	14 (18.2)	12 (15.6)	8 (10.4)	5 (6.5)	* 0.25 (0.10–0.40)
	MR	69 (89.6)	1 (1.3)	3 (3.9)	3 (3.9)	1 (1.3)	
R.IPS	CT	57 (74.0)	12 (15.6)	5 (6.5)	1 (1.3)	2 (2.6)	0.06 (−0.10~0.23)
	MR	66 (85.7)	6 (7.8)	2 (2.6)	2 (2.6)	1 (1.3)	
R.PP	CT	59 (76.6)	11 (14.3)	2 (2.6)	2 (2.6)	3 (3.9)	* 0.23 (0.06–0.40)
	MR	68 (88.3)	4 (5.2)	2 (2.6)	2 (2.6)	1 (1.3)	
L.CS	CT	18 (23.4)	23 (29.9)	8 (10.4)	19 (24.7)	9 (11.7)	0.07 (−0.06–0.20)
	MR	57 (74.1)	7 (9.1)	4 (5.2)	3 (3.9)	6 (7.8)	
L.SPS	CT	37 (48.1)	12 (15.6)	12 (15.6)	9 (11.7)	7 (9.1)	0.16 (0.01–0.30)
	MR	66 (85.7)	6 (7.8)	1 (1.3)	2 (2.6)	2 (2.6)	
L.IPS	CT	57 (74.0)	12 (15.6)	3 (3.9)	2 (2.6)	3 (3.9)	0.13 (−0.08–0.35)
	MR	59 (76.6)	7 (9.1)	7 (9.1)	3 (3.9)	1 (1.3)	
L.PP	CT	56 (72.7)	10 (13)	5 (6.5)	4 (5.2)	2 (2.6)	* 0.30 (0.10–0.51)
	MR	61 (79.2)	3 (3.9)	5 (6.5)	5 (6.5)	3 (3.9)	

Notes: Data represent the number of cases. * There is minimal statistical agreement. CTA, computed tomography angiography; TOF-MRA, magnetic resonance angiography; CT, computed tomography; MR, magnetic resonance; CS, cavernous sinus; SPS, superior petrosal sinus; IPS, inferior petrosal sinus; PP, pterygoid plexus; CI, confidence interval; R, right; L, left.

## Data Availability

The data that support the findings of this study are available on reasonable request from the corresponding author. The data are not publicly available due to privacy or ethical restrictions.

## Appendix A

**Table A1:** Frequency of flow-related signal intensity in CS, SPS, IPS, and PP by grading in 77 participants (cohort 2).

Modality	Area	5-Point Scale					*p* Value *
		Grade 0 (%)	Grade I (%)	Grade II (%)	Grade III (%)	Grade IV (%)	
MR	R.CS	57 (74.0)	11 (14.3)	2 (2.6)	6 (7.8)	1 (1.3)	0.573
	L.CS	57 (74.0)	7 (9.1)	4 (5.2)	3 (3.9)	6 (7.8)	
	R.SPS	69 (89.6)	1 (1.3)	3 (3.9)	3 (3.9)	1 (1.3)	0.313
	L.SPS	66 (85.7)	6 (7.8)	1 (1.3)	2 (2.6)	2 (2.6)	
	R.IPS	66 (85.7)	6 (7.8)	2 (2.6)	2 (2.6)	1 (1.3)	0.108
	L.IPS	59 (76.6)	7 (9.1)	7 (9.1)	3 (3.9)	1 (1.3)	
	R.PP	68 (88.3)	4 (5.2)	2 (2.6)	2 (2.6)	1 (1.3)	0.095
	L.PP	61 (79.2)	3 (3.9)	5 (6.5)	5 (6.5)	3 (3.9)	

Notes: Data represent the number of cases. * Wilcoxon test was used to evaluate whether the finding was more frequent on the left or right side. However, the difference in frequency was not statistically significant. MR, magnetic resonance; CS, cavernous sinus; SPS, superior petrosal sinus; IPS, inferior petrosal sinus; PP, pterygoid plexus; R, right; L, left.
